# Analysis of Differentially Expressed Proteins in* Mycobacterium avium*-Infected Macrophages Comparing with* Mycobacterium tuberculosis*-Infected Macrophages

**DOI:** 10.1155/2017/5103803

**Published:** 2017-05-10

**Authors:** Dongjun Yang, Xin Fu, Shiyi He, Xueping Ning, Min Ling

**Affiliations:** Department of Biochemistry and Molecular Biology, Guangxi Medical University, Key Laboratory of Biological Molecular Medicine Research of Guangxi University, Nanning, Guangxi 530021, China

## Abstract

*Mycobacterium avium* (MA) belongs to the intracellular parasitic bacteria. To better understand how MA survives within macrophages and the different pathogenic mechanisms of MA and* Mycobacterium tuberculosis* (MTB), tandem mass tag (TMT) and liquid chromatography-tandem mass spectrometry (LC-MS/MS) analysis have been used to determine the proteins which are differentially expressed in MA-infected and MTB-infected macrophages. 369 proteins were found to be differentially expressed in MA-infected cells but not in MTB-infected cells. By using certain bioinformatics methods, we found the 369 proteins were involved in molecular function, biological process, and cellular component including binding, catalytic activity, metabolic process, cellular process, and cell part. In addition, some identified proteins were involved in multiple signaling pathways. These results suggest that MA probably survive within macrophages by affecting the expression of some crucial proteins.

## 1. Introduction

Tuberculosis (TB) is an infectious disease which is transmitted in the air and caused by bacillus* Mycobacterium tuberculosis *(MTB) infection, making it a major global health problem. MTB could also be largely found in patients who were affected by HIV [1, 2].* Mycobacterium avium *(MA), one of the* Nontuberculous Mycobacteria *(NTM), is a ubiquitous organism usually isolated from natural environments such as soil and drinking water and causes infections such as disseminated disease, bacteremia, and severe pulmonary in immunocompromised people, especially in patients who suffered from AIDS [3, 4]. Besides MA, there are 4 different types of NTM that could also cause lung disease including* mycobacterium kansasii, mycobacterium abscessus, mycobacterium xenopi, *and* mycobacterium malmoense *[5, 6].

Macrophage serves as the first defense response of a host to MTB or MA infections [7]. Several membrane receptors, including CD14, CD43, Toll-like receptors (TLRs), complement receptors, mannose receptors, and scavenger receptors, are involved in the integration of MTB or MA with macrophage [8–12]. Uptake of MTB or MA by macrophage triggers a series of cell signaling pathways and initiation of an immune response. However, the mechanism of MTB- and MA-induced macrophage infection is different. MTB often leads to arrest of phagosome maturation, antiapoptosis response, and suppression of antibacterial response, while MA infection induces antibacterial response and phagosome maturation [4, 13–15]. Recent studies have shown that the patient who suffers from organ transplant, immunodeficiency, and gene heterozygote of Cystic Fibrosis Transmembrane Regulator (CFTR) is more easily infected by MA than normal people. And the one who has genetic defects of (interferon) IFN-*γ* and (interleukin) IL-12 pathway is more susceptible to MA infection as well [4, 6]. Similar to patients infected with MTB, patients with MA infection are often treated with (tumor necrosis factor) TNF-*α* blocker [4, 6] whose increased risk is less well defined and must be used with greater caution.

MTB and MA are facultative pathogens capable of growing inside macrophages. The macrophage probably exerts very strong selective pressure on the mycobacteria residing within it, influencing the expression of gene products essential for the survival of the bacteria within this hostile environment. The induction of survival mechanisms, alongside a range of immunological effector molecules, emphasizes the complexity of the cross-talk that occurs between the macrophage and the mycobacterium. To characterize this cross-talk and to detail the changes which occur following the initial interaction between mycobacterium and macrophage, some researchers have studied the proteins changes of the host cell in the early stages of mycobacterial infection for a long time [16–19]. However, they often used RT-PCR, ELISA, or MS to study the individual or several related proteins of the cells. And changes in protein profiles of cells infected with mycobacterium, especially with MA infection, are still poorly understood.

In this study, we used a method called TMT technology [2], which shared the same principle of iTRAQ, to identify the differentially expressed proteins which were produced by a human monocyte cell line U937 infected with MTB and MA, respectively. By using proteomics analysis methods, we investigated the identified proteins which are differentially expressed in MA-infected but not in MTB-infected cells and the biological processes and signaling pathways these proteins were involved in. Identification of proteins by such a strategy will enable us to extend our understanding of the cross-talk between intracellular pathogens and their host cells and identify novel mechanisms of bacterial evasion or immunological elimination which can give us some suggestions on a therapeutic approach to fight against MA infection.

## 2. Materials and Methods

### 2.1. Cell Culture and Infection

The MTB and MA were obtained from Center for Disease Prevention and Control of Guangxi. The U937 cell line was obtained from Obio Technology (Shanghai) Co. Ltd. U937 cell line was cultured at 37°C, 5% CO_2_ in RPMI 1640 medium supplemented with 10% fetal bovine serum, penicillin (100 *μ*g/mL), and streptomycin (30 *μ*g/mL) until it was grown to a density of 1 × 10^7^ cells per mL. Subsequently, cells were centrifuged, washed, and resuspended in RPMI 1640 supplemented with 10% fetal bovine serum. The cell density was adjusted to 10^6^ cells per mL and 2 mL of the cell suspension was transferred to a 6-well tissue culture plate. MTB was cultured in Lowenstein-Jensen culture medium and MA was cultured in Egg culture medium. Both bacteria were cultured for 15 days and then harvested, washed, and suspended in phosphate buffer saline. The suspension was diluted to a concentration of 1 × 10^8^ CFU/mL to make sure the MOI was 1 : 100. The treated MTB and MA were added to 6-well tissue culture plate which was precultured with U937 cells. The cells were divided into three groups: cells infected with MTB, cells infected with MA, and cells with PBS (control). The cells of each group were cultured at 37°C, 5% CO_2_ for 24 hours, respectively.

### 2.2. Sample Preparation

The cells of three groups were centrifuged at 4°C, 1000*g* for 20 minutes. The supernatant was discarded. The cell pellets were lysed in lysis buffer containing protease inhibitor mix and then centrifuged at 4°C, 16000*g* for 15 mins. The supernatant was transferred to a precooled centrifugal tube. The cell lysate was stored at −80°C until further analysis.

### 2.3. Protein Quantification and Extraction

Bradford method was used to measure the protein concentrations. For each sample, 100 *μ*g of protein was transferred to a new microcentrifuge tube and adjusted to a final volume of 100 *μ*L with 100 mM TEAB. 5 *μ*L of 200 mM TCEP was added and incubated with the sample at 55°C for 1 hour. Another 5 *μ*L of 375 mM iodoacetamide was added and incubated with the sample mixture for 30 mins in the dark at room temperature. At last, 600 *μ*L of cold acetone was used to precipitate proteins at −20°C for about 4 hours. Precipitated protein was centrifuged at 8000*g* for 10 mins at 4°C and then was dried and stored at −80°C until further use.

### 2.4. Protein Digestion and Peptide Labeling

The protein pellet was resuspended with 100 *μ*L of 100 mM TEAB and incubated with 20 *μ*L of the Trypsin Storage Solution for 5 mins. 2.5 *μ*L of trypsin was used to digest 100 *μ*g of protein of each sample overnight at 37°C. The protein sample was dissolved in anhydrous acetonitrile with occasional vortexing. TMT labeling reagent was added and incubated with the sample for 1 hour at room temperature. MA-infected group sample was labeled with reagent 127 and MTB-infected group sample was labeled with reagent 128 while the sample of control group was labeled with reagent 126. 5% hydroxylamine was used to quench the reaction. The labeled samples were stored at −80°C until future analysis.

### 2.5. HPLC (High Performance Liquid Chromatography) Separation of Peptide Mixture

Firstly, we separated polypeptide with C18 reverse phase column. Protein samples labeled with reagents 126, 127, and 128 were air-dried with vacuum followed by enzyme digestion. The peptide mixture was acidified with mobile phase A (10 mM KH_2_PO_4_, 20% v/v acetonitrile, and pH 3.0) and then loaded onto polyethylene A. The peptide mixtures were eluted with a flow rate of 700 *μ*L/min and a gradient of 0–40% mobile phase B (mobile phase A plus 500 mM KCl) for 50 minutes, 40% B–100% B for 10 minutes, 100% B–100% B for 5 minutes, 100% B–100% A for 5 minutes, and 100% A for 10 minutes. The eluates were monitored by absorbance at a wavelength of 254 nm, and the fractions were collected every 2 minutes. The collected fractions of 28 tubes were desalted on C18 reverse phase column.

### 2.6. LC-MS/MS Analysis and Quantification

Each fraction underwent nano-LC-MS/MS analysis using a Q Exactive MS equipped with Easy nLC. The chromatographic column was balanced with mobile phase A (2% ACN + 0.1% HCOOH). The peptide mixtures were separated at a flow rate of 300 *μ*L/min. Mobile phase B (98% ACN + 0.1% HCOOH) was also used and the elution gradient was 95% A for 5 minutes, 95%–65% A for 45 minutes, 35% A–100% B for 2 minutes, and 100% B for 3 minutes. The mass spectrometer data were analyzed using the ESI in the positive ion mode with a selected mass range of 350–2000 mass/charge (*m*/*z*) and the survey scans were acquired at a resolution of 60000. The resolution for HCD spectra was set at 15000. Normalized collision energy was 1.5 KV with a collision mode of CID. The instrument was set at data-dependent automatic collecting mode.

### 2.7. Proteomic Data Analysis

The primitive data collected by MS was searched and matched with SEQUEST searching material of Proteome Discovery software which uses Xoc BLS256 database of NCBI as its main database. The settings were as follows: trypsin chosen as the enzyme with one missed cleavage allowed; fixed modifications of carbamidomethylation at Cys; variable modifications of oxidation at Met; peptide tolerance set at 0.05 Da and MS/MS tolerance at 0.1 Da; and monoisotopic mass chosen. Proteins with 2-fold or greater and 0.5-fold or less changes between successive comparisons with a *P* value less than 0.05 were determined as significantly differentially expressed [20].

### 2.8. Enrichment of GO and KEGG Pathways

We searched the GO and KEGG database to classify and identify differentially expressed protein. The significant signaling pathway enrichment was examined with the hypergeometric test. A *P* value < 0.05 was considered statistically significant.

## 3. Results and Discussion

### 3.1. Identification of Differentially Expressed Protein

In this study, U937 cells without infection (control) and U937 cells infected with MA and MTB culture, respectively, for 24 hours were collected for protein extraction, digestion, and TMT labeling. Proteomes of both infections were investigated.

In the sample of MA- and MTB-infected U937 cells, 2269 proteins were identified. Among these proteins, a total of 574 proteins were differentially expressed in MA-infected cells and 887 proteins in MTB-infected cells (versus control, changes ≥2.0- or ≤0.5-fold, *P* value < 0.05). Compared to the identified proteins of MA- and MTB-infected groups, we found that 369 proteins were differentially expressed in MA-infected but not in MTB-infected cells. Among them, 2 proteins were upregulated (≥2.0-fold, *P* value < 0.05) while 367 proteins were downregulated (≤0.5-fold, *P* value < 0.05). 682 proteins were differentially expressed in MTB-infected but not in MA-infected cells. Among them, 672 proteins were upregulated (≥2.0-fold, *P* value < 0.05) while 10 proteins were downregulated (≤0.5-fold, *P* value < 0.05).

### 3.2. GO-Annotation

To understand the difference between MA infection and MTB infection, the 369 proteins which were only differentially expressed in MA-infected cells are characterized with GO term (http://www.geneontology.org/).

**Figure 1:**
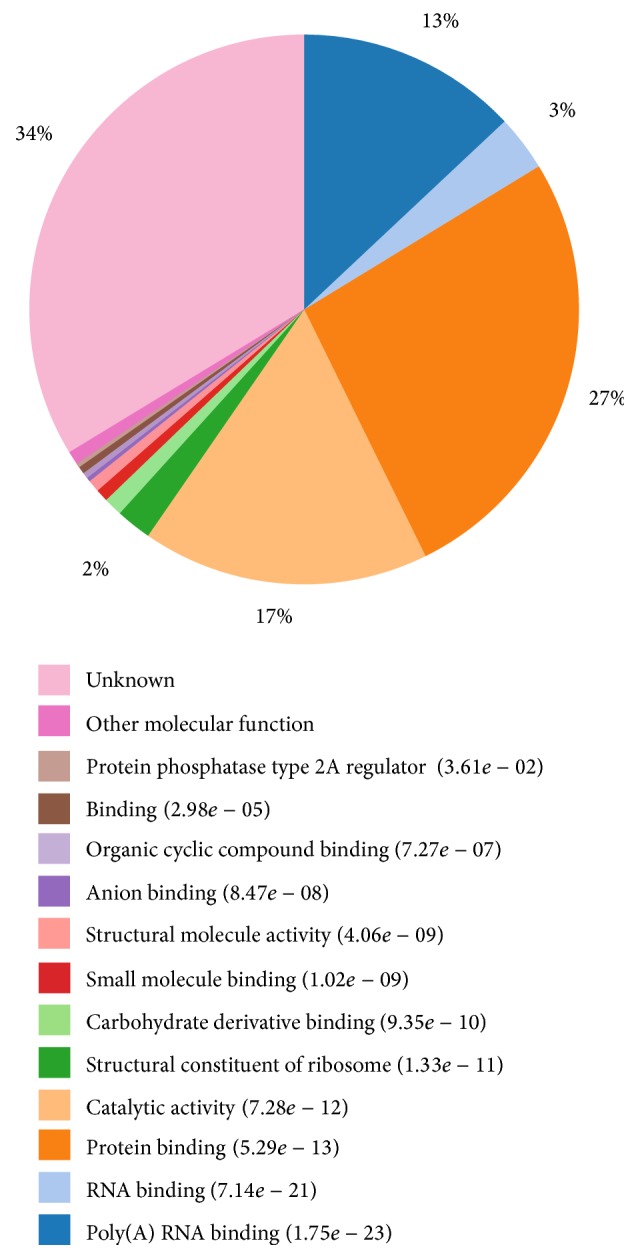
GO terms distribution in molecule function. The numbers in parenthesis are *P* value (note: the less the *P* value, the better the result of the clustering of GO terms).

The identified 369 proteins can be categorized into three functional groups: molecular function, biological process, and cellular components. The proteins identified in molecular function were found to be mainly involved in protein binding (27%), catalytic activity (17%), and Poly(A) RNA binding (13%) (Figure 1). In terms of protein binding, some proteins were involved in calcium ion binding and calcium-dependent phospholipid binding. For example, calmodulin 1 (CAM1), calmodulin 2 (CAM2), and calmodulin 3 (CAM3) were identified calcium ion binding proteins. Calcium-binding protein 39 (Q9Y376) was involved in calcium-dependent phospholipid binding. CAMs and PPP3R1 would affect the proliferation and fertilization of the cell by binding to calcium ion. In addition, the protein DNA fragmentation factor (DFFA) was found in the calcium signaling pathway during protein processing in endoplasmic reticulum [21]. Some proteins were new receptors which were involved in MA interaction with macrophage. For example, FCAR was associated with immunoglobulin alpha Fc receptor and SNED1. Q92478 was related to EGF-like domain and C-type lectin domain which were parts of many classic receptors. The identified proteins were also involved in the activity of many enzymes, the majority of which were deaminase, enzyme regulator, helicase, hydrolase, isomerase, ligase, oxidoreductase, and transferase. All these enzymes were associated with DNA replication, protein processing, metabolism, and other biological process.

**Figure 2:**
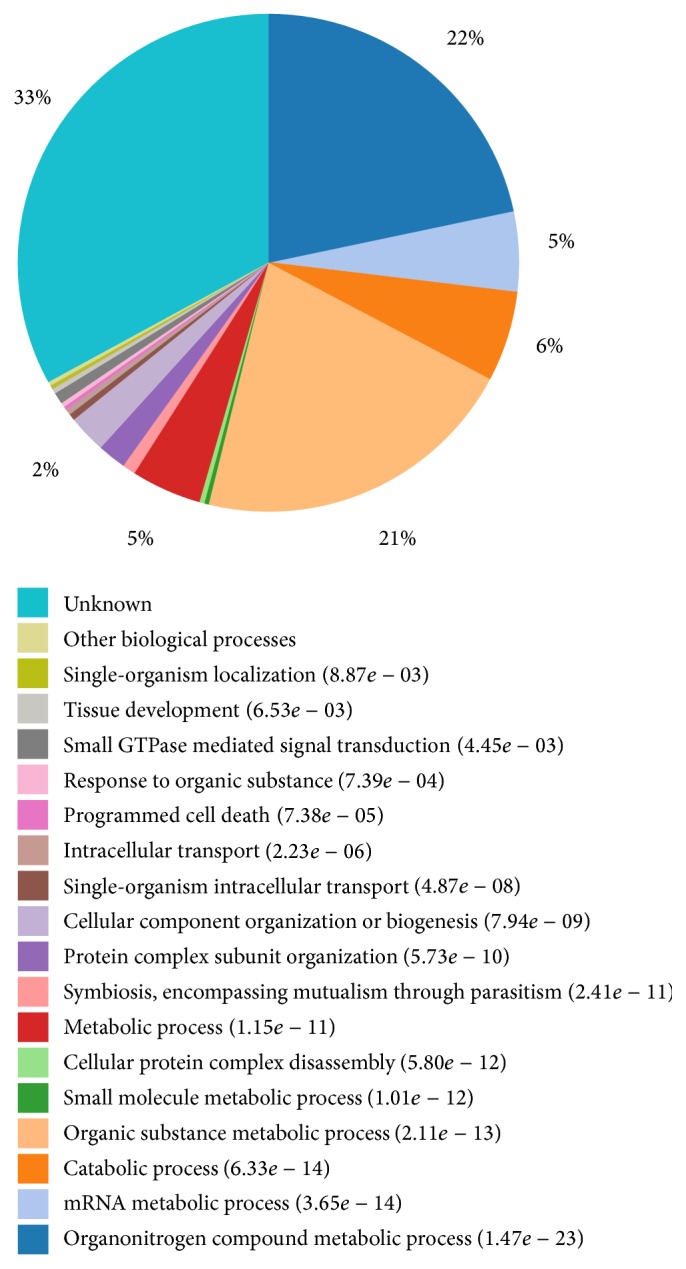
GO terms distribution in biological process. The numbers in parenthesis are *P* value (note: the less the *P* value, the better the result of the clustering of GO terms).

The proteins identified in biological process were found to be mostly involved in organonitrogen compound metabolic process (22%), organic substance metabolic process (21%), and catabolic process (6%) (Figure 2). The identified proteins were mainly involved in cell communication and cell cycle of meiosis and mitosis. Several proteins were related to cell cycle pathway and other pathways. BUB3 (mitotic checkpoint protein) was the DNA damage checkpoint protein, which could control cell cycle and cell proliferation and also induce cell apoptosis by affecting the regulation of protein p53 [22]. Smc1A (structural maintenance of chromosomes 1A) was associated with ubiquitin-mediated proteolysis [23]. CDK1 and CycB could mediate the cell division from G1 to S and G2 to M phases [24]. Rb1 (retinoblastoma-associated protein 1) and HDAC2 (histone deacetylase 2) were involved in apoptosis, S-phase, and DNA biosynthesis [25].

**Figure 3:**
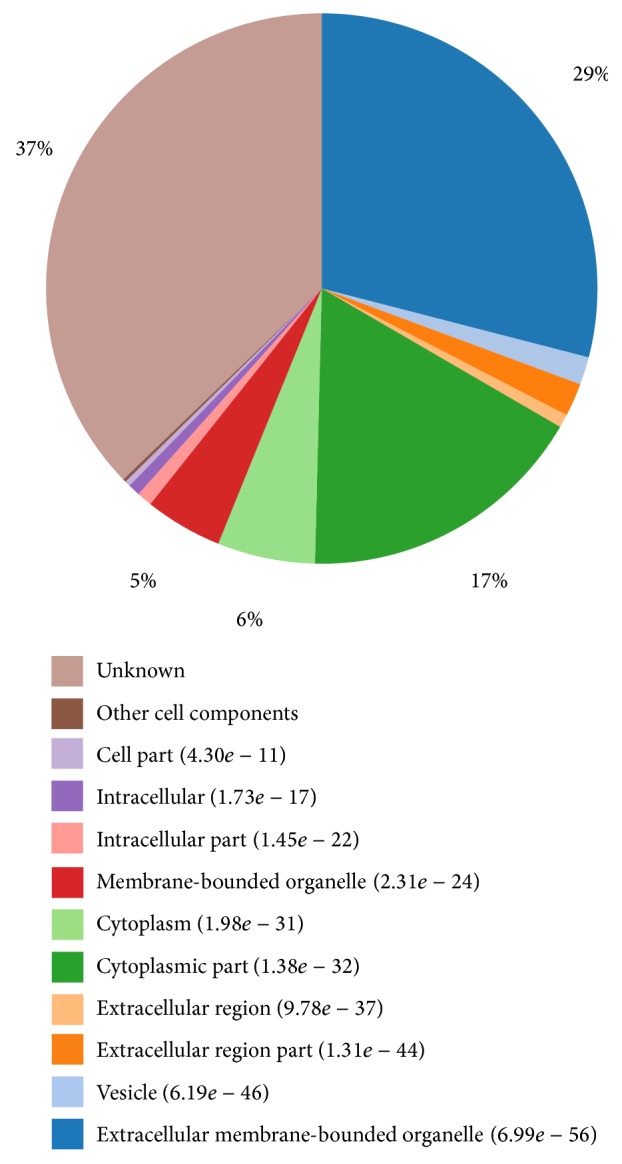
GO terms distribution in cellular component. The numbers in parenthesis are *P* value (note: the less the *P* value, the better the result of the clustering of GO terms).

Among proteins classified as cellular component, the majority was localized in extracellular membrane-bounded organelle (29%) and cytoplasmic part (17%) (Figure 3). Most of the identified extracellular membrane-bounded organelle proteins such as TUBA1B, ITGB2, UBA1, and ACTB were localized to intracellular and plasma membrane, which suggested that MA has invaded into the cell by attaching to the cell membrane and then affected the expression of the extracellular protein. MA also affected the regular expression of the protein of cytoplasmic part such as P07858, Q9Y2R5, and MRPS17.

It is notable that the only two proteins named P10412 and H0YJ03 were upregulated in MA-infected cells compared to MTB-infected cells. P10412 is histone H1.4 and is necessary for the condensation of nucleosome chains into higher-order structured fibers. It also regulates chromatin remodeling, DNA methylation, and many other functions [26]. H0YJ03 is proteasome subunit alpha type 3. Proteasomes are distributed at a high concentration in eukaryotic cells and cleave peptides in an ATP/ubiquitin-dependent process in a nonlysosomal pathway [27].

### 3.3. KEGG Pathway Analysis

We used DAVID Functional Annotation Bioinformatics Microarray Analysis (https://david.ncifcrf.gov) and Omicsbean (http://www.omicsbean.cn) to analyze the 369 proteins which were differentially expressed in MA infection but not MTB infection. Hopefully our findings would identify in which pathway these proteins were involved in order to understand the mechanism of MA infection into macrophages. The analysis came up with 152 KEGG orthologues. All these signaling pathways may suggest how MA affects the expression of proteins in MA-infected cells. The KEGG pathways with more differentially expressed proteins involved included Hippo signaling pathway, PI3K-Akt signaling pathway, and phagosome pathway.

#### 3.3.1. The Hippo Signaling Pathway

**Figure 4:**
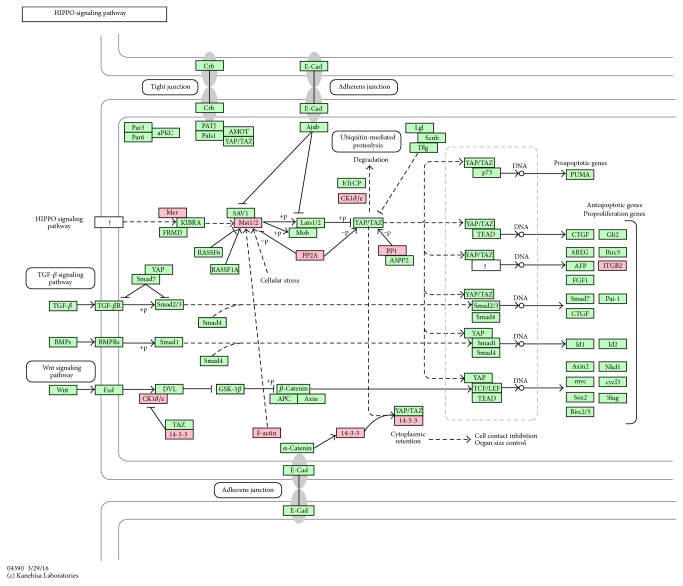
KEGG pathway for Hippo signaling pathway. The differentially expressed proteins were labeled as red.

10 differentially expressed proteins (YWHAQ (14-3-3), YWHAH (14-3-3), ITGB2 (ITGB2), ACTB (F-actin), PPP2R2A (PP2A), STK3 (Mst1/2), PPP1CA (PP1), CSNK1E (CK1*δ*/*ε*), NF2 (Mer), and PPP2R1A (PP2A)) were associated with Hippo signaling pathway (Figure 4). The Hippo signaling pathway, which is also called the Salvador/Warts/Hippo (SWH) pathway, regulates the proliferation and apoptosis of cells and controls the size of animal organ. When the Hippo signaling pathway is activated, the cell proliferation is arrested and the cell apoptosis is induced. Thus this signaling pathway is most prominent and extremely important in the study of human cancer [28]. This signaling pathway starts with the phosphorylation of the protein kinase Warts (Wts) [29] which is activated by Hpo, a core kinase cascade leading to activating STK3. STK3 is a member of Ste-20 family protein kinase. This serine/threonine kinase regulates several cellular processes including the proliferation, growth, and apoptosis of cells, cell cycle progression, and response to stress. The transmembrane protein Fat and other membrane associated proteins such as NF2, a FERM domain-containing apical proteins, act as upstream regulators of the core Hpo/Wts kinase cascade. NF2 was also identified in our study suggesting that MA may survive inside macrophage by preventing apoptosis and affecting the cell proliferation with a mechanism of disturbing the regular expression of these core proteins.

Other proteins such as PPP2R2A, PPP1CA, CSNK1E, and PPP2R1A are involved in the Hippo signaling pathway as the downstream regulator of the core Hpo/Wts kinase cascade, while YWHAQ, YWHAH, ITGB2, and ACTB lead to cell contact inhibition and organ size control.

#### 3.3.2. The PI3K-Akt Signaling Pathway

**Figure 5:**
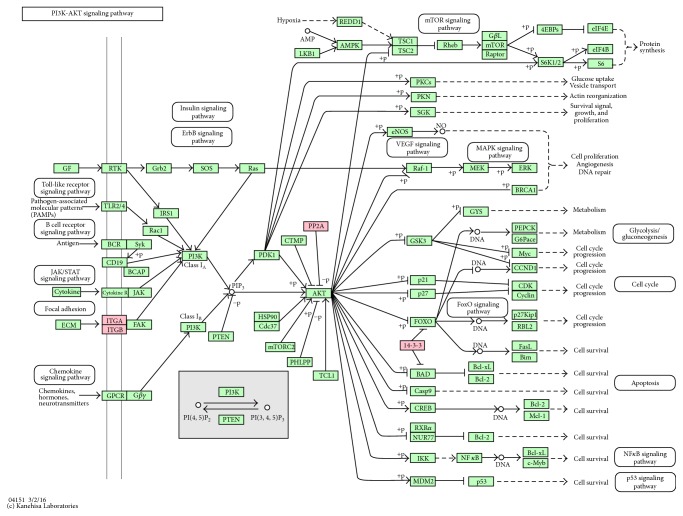
KEGG pathway for PI3K-Akt signaling pathway. The differentially expressed proteins were labeled as red.

6 identified proteins are involved in the PI3K-Akt signaling pathway, which are YWHAQ (14-3-3), YWHAH (14-3-3), ITGA5 (ITGA), ITGB1 (ITGB), PPP2R2A (PP2A), and PPP2R1A (PP2A) (Figure 5). The PI3K-Akt signaling pathway is a typical intracellular signaling pathway regulating the cell cycle, cell proliferation, cells apoptosis, and some metabolic process such as glycolysis [30, 31]. Akt is activated and phosphorylated by PI3K, which leads to activation of downstream effectors like CREB, Ptdlns-3ps, and mTOR. It could also inhibit the expression of p27. As shown in Figure 5, ITGA5 and ITGB1 are integrin subunits. They interact with FAK (focal adhesion kinase) to regulate the expression of PI3K and Akt. On the contrary, the serine/threonine-protein phosphatase PPP2R2A and PPP2R1A types dephosphorylate Akt which results in the activation of apoptosis. Our study found both PPP2R2A and PPP2R1A were downregulated, which led to reduced dephosphorylation of Akt and subsequent inhibition of apoptosis. So MA can survive inside macrophage by regulating the expression of these proteins.

#### 3.3.3. The Phagosome Signaling Pathway

**Figure 6:**
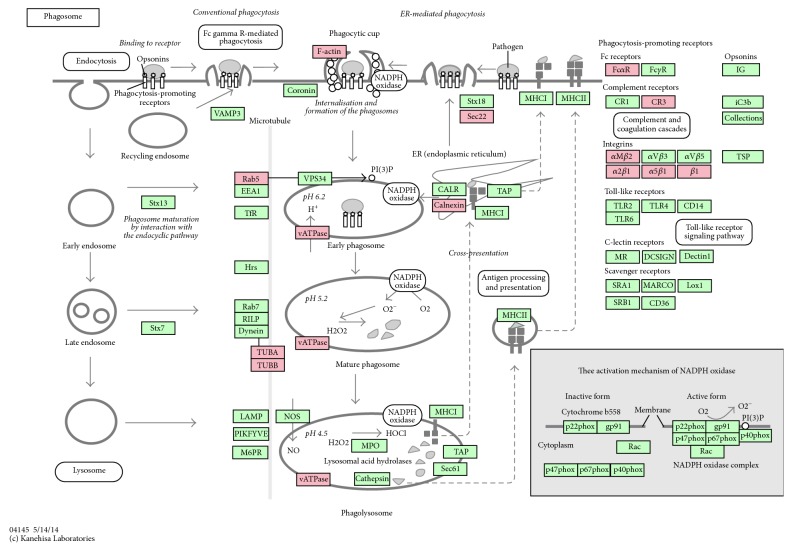
KEGG pathway for phagosome. The differentially expressed proteins were labeled as red.

Phagosome is a vesicle which is absorbed by cells via phagocytosis. It is also a cellular compartment which could fuse with lysosomes in the process of maturation to form phagolysosomes to kill and digest pathogenic microorganisms. After invading into cells, some pathogenic bacteria could reproduce inside a formed phagolysosome or escape into the cytoplasm just before the phagosome fuses with lysosomes [32]. Many mycobacteria like MTB could manipulate the macrophage of the host to prevent lysosomes from fusing with phagosomes and creating mature phagolysosomes, thus creating a comfortable environment for the pathogens inside it [33, 34]. 12 proteins were identified, associated with this cell process, which were SEC22B (Sec22), TUBB (TUBB), ITGA5 (*α*5*β*1), ACTB (F-actin), ITGB2 (CR3), ITGB1 (*β*1), TUBA1B (TUBA), ATB6V1B2 (vATPase), SEC22B (Sec22), CANX (calnexin), RAB5A (Rab5), and FCAR (FcaR) (Figure 6). FCAR is one of the Fc receptors. ITGB2 and ITGB1 are complement receptors, both of which belong to phagocytosis-promoting receptors and are involved in the apoptosis of cells. CANX is associated with antigen presentation which has a significant role in immune response on the cell surface. RAB5A and ATB6V1B2 are associated with the formation of phagosome when it changes from early phagosome to mature phagosome and then fuse with lysosome to become phagolysosome. These events are connected with acidification of cellular inner environment and an increase in intracellular calcium ion concentration which are essential for destruction of mycobacteria [35]. TUBA1B and TUBB are major constituent of microtubules. They bind with GTP and function in many processes, including structural support, intracellular transport, and DNA segregation of phagosome.

## 4. Conclusions

In summary, we carried out a proteomic analysis of differentially expressed proteins with TMT technology in MA-infected macrophage. It is the first study to analyze the proteins involved in MA infection. In addition, we profiled the differentially expressed proteins in MTB infections. By comparing the proteomic data of the two different infections, we were able to identify the 369 differentially expressed proteins which were unique in MA-infected cells. These identified proteins were involved in binding, catalytic activity, metabolic process, cellular process, cell apoptosis, phagosome maturation, antimycobacterial response, cellular components, and so forth. In addition, some identified proteins were involved in multiple signaling pathways. The results of this study will help elucidate how MA survives within cells and the different pathogenic mechanisms of MA and MTB infections. It might also provide new ideas for future development of target treatment of MA disease and tuberculosis.
